# *SHE9* deletion mutants display fitness defects during diauxic shift in *Saccharomyces cerevisiae* .

**DOI:** 10.17912/micropub.biology.000899

**Published:** 2023-07-28

**Authors:** Shane J. Kowaleski, Christina S. Hurmis, Carvin N. Coleman, Kieli D. Philips, Nicole A. Najor

**Affiliations:** 1 Biology Department, University of Detroit Mercy

## Abstract

*Saccharomyces cerevisiae* protein She9 is localized to the inner mitochondrial membrane and is required for normal mitochondrial morphology. While deletion mutants of *SHE9* ( *she9Δ* ) are viable and display large ring-like mitochondrial structures, the molecular function of *SHE9* is still unknown. We report a decreased growth of *she9Δ* cells during a diauxic shift, where mitochondria are primarily employing oxidative phosphorylation to generate ATP versus the alternative mechanism of glycolysis in high glucose conditions. Further bioinformatics analysis reveal putative functional protein associations, and proposes a model to aid in the understanding of the molecular function of She9.

**Figure 1. Decreased growth of she9Δ on a non-fermentable carbon source:**
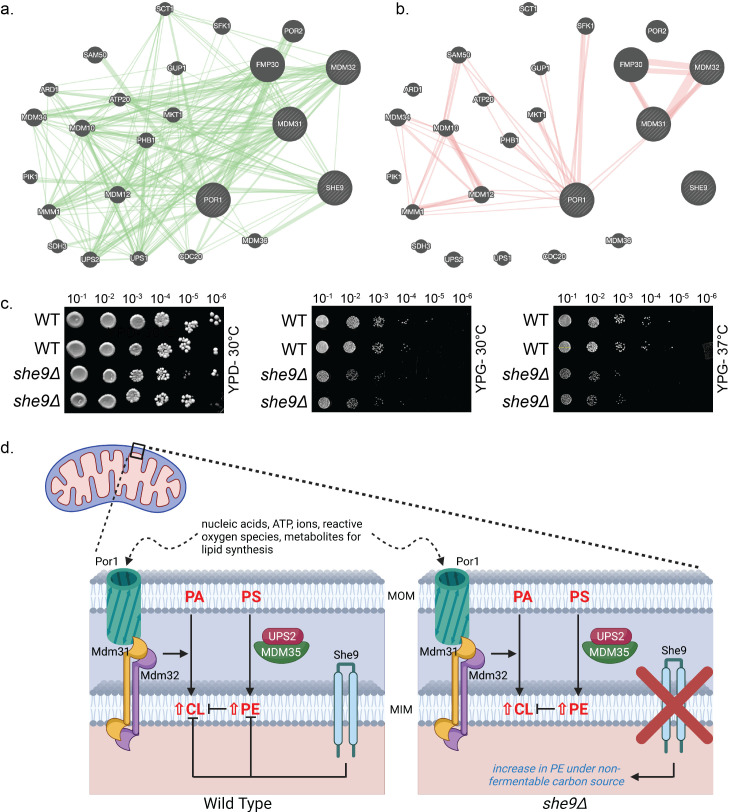
(A) GeneMANIA (www.genemania.org) was used to create a genetic interaction network whereby green lines represent whether two genes have been shown to be functionally associated. (B) GeneMANIA was used to generate a physical interaction network whereby red lines represent if gene products are linked in protein-protein interaction studies. (C) Yeast deleted of *SHE9 (she9* ***Δ*** ) were grown on YPG plates after preculture on YPD. Wild type (WT) strains are the BY4741 strain background. The plates were incubated at 30°C and 37°C for 2-3 days. Biological triplicates are from independent single colonies streaked from the same original stock either on YPD for WT and YPD + G418 plates for *she9* ***Δ*** . Representative experiment is shown. Serial dilutions are as indicated. (D) Model describing putative molecular function of She9 (created with BioRender.com). Abbreviations: phosphatidic acid (PA), phosphatidylserine (PS), cardiolipin (CL), phosphatidylethanolamine (PE), mitochondrial outer membrane (MOM), mitochondrial inner membrane (MIM).

## Description

Mitochondria are organelles that carry out essential metabolic pathways in a diversity of species and organisms. Proper mitochondrial functioning affects mechanisms that drive developmental processes, aging, and apoptosis. Since mitochondria do not undergo *de novo* synthesis, they must be inherited, and many studies have intricately described the fusion and fission events that ensure proper generation of mitochondria. There are three conserved mitochondrial dynamin-related GTPases in yeast that mediate fusion and fission events of mitochondrial dynamics; Fzo1, Mgm1 and Dnm1 (Fritz et al. 2001; van der Bliek et al. 2013; Zhang et al. 2014) . Mitochondrial fusion and fission occur at similar rates, and assist in promoting overall homeostasis of mitochondria. During mitochondrial fusion, individual mitochondria will fuse to promote the merging of the contents with those fused mitochondria. The protein Fzo1 is localized to the mitochondrial outer membrane (MOM) assisting with the fusion of adjacent MOMs, while the protein Mgm1 is localized to the mitochondrial inner membrane (MIM), assisting with the fusion of adjacent MIMs, respectively. In addition to these membrane specific proteins, there are other adaptor proteins in-play that are critical to assisting the coordination of fusing both the MOM and the MIM (Fritz et al. 2001; van der Bliek et al. 2013) .

Unlike the fusion machinery, which has components at the MOM and MIM, mitochondrial fission largely depends on one dynamin-related GTPase, specifically Dnm1. Dnm1 assembles on the surface of mitochondria at sites identified by a platform of proteins (Elgass et al. 2013; Kraus et al. 2021) . Interestingly, in addition to the dynamin-related GTPase proteins important for mitochondrial fusion and fission, there is evidence that supports the role of phospholipids in mitochondrial dynamics through interactions with the dynamin GTPases. Specifically, proper phospholipid composition was shown to be required for biogenesis, assembly, and activity of the dynamin-related GTPases (Osman et al. 2011) . The mitochondrial membrane synthesizes certain phospholipids after import of their precursors from the endoplasmic reticulum (ER). The mitochondrial membranes consist of phosphatidylcholine (PC), phosphatidylethanolamine (PE), phosphatidylinositol (PI), phosphatidylserine (PS), phosphatidic acid (PA), and a mitochondrial-specific phospholipid cardiolipin (CL) (Zinser et al. 1991; de Kroon et al. 1999; van Meer et al. 2008; Horvath and Daum 2013) . It is known that mitochondrial synthesis of CL and PE first requires the transport of precursors phosphatidic acid (PA) and phosphatidylserine (PS) into the mitochondria. This begins with transport of PA and PS from the ER to the MOM through membrane tethering complexes forming physical contact sites (Kornmann et al. 2009; Lahiri et al. 2014) . PA and PS are then transferred to the MIM through intermembrane space protein complexes, Ups1-Mdm35 and Ups2-Mdm35, where they are then converted to PE and CL (Connerth et al. 2012; Watanabe et al. 2015; Aaltonen et al. 2016; Miyata et al. 2016) .

Both mitochondrial fusion and fission are important processes required for inheritance upon cell division, mitochondrial redistribution, and maintenance of a healthy mitochondrial network. In addition, both processes have been shown to play prominent roles in disease related processes of cancer, autosomal dominant optic atrophy (ADOA), Charcot-Marie-Tooth neuropathy 2A, Behr syndrome, Parkinson's disease, Alzheimer’s disease, Huntington’s disease, and a variety of neuropathic, myopathic, ataxic, and atrophic disorders (Calzada et al. 2016; Serasinghe and Chipuk 2017; Al Ojaimi et al. 2022) . Considering the devastation that can be caused by mitochondrial fission and fusion disruption, it is clear that full understanding of the proteins involved in these mechanisms will aid in further understanding of these disorders as well as providing potential new therapeutic targets.

In a genome-wide screen to identify genes important for mitochondrial distribution and morphology, *SHE9* (also known as *MDM33* ) was identified. She9 is localized to the MIM, and is proposed to dimerize through its coiled-coil domains (Messerschmitt et al. 2003) . In an analysis of epitope tagged versions of *SHE9,* researchers found that the C-terminus was required to rescue defects caused by the deletion of *SHE9 (she9Δ)* (Messerschmitt et al. 2003) . Additionally, *she9Δ* cells exhibit large ring-like mitochondrial structures (Dimmer et al. 2002) and extremely long mitochondria that would extend through half of the cell (Messerschmitt et al. 2003) . Interestingly, *she9Δ* cells are able to undergo mitochondrial fusion, which is the proposed mechanism by which the large ring-like structures are formed in the deletion mutants (Messerschmitt et al. 2003) , but show decreased mitochondrial fission activity (Klecker et al. 2015) . Conversely, when *SHE9* was overexpressed, the mitochondria formed membranous partitions/septa that separated the inner compartment into distinct chambers (Klecker et al. 2015) , and some mitochondria were reported to be largely devoid of cristae (Messerschmitt et al. 2003; Klecker et al. 2015) . Overexpression experiments of *SHE9* also cause a decrease in both phospholipids CL and PE, as detected by mass spectrometry (Klecker et al. 2015) . Studies have also found that *she9Δ* mutants are epistatic to many genes encoding mitochondrial structure and dynamics, except for the deletion of mitochondrial genes *FMP30, GEM1, MDM10, MDM12, MDM31, MDM34, or MMM1* , where in these double mutants the phenotype due to *she9Δ* was largely lost (Klecker et al. 2015) . Interestingly, all of these genes are involved in lipid metabolism. While a wealth of research has uncovered morphological defects of loss or gain of function of *SHE9,* its molecular function has yet to be solved.

To better understand the molecular function of She9 a number of bioinformatics analyses were performed through open-access databases. A string analysis through GeneMANIA (www.genemania.org) identified specific genetic (green lines) and physical interactors (red lines) of *SHE9* /She9 ( Figure 1a, 1b). A number of potential players were identified, but attention was drawn to MIM proteins Mdm31/Mdm32 and MOM protein Por1, which have been shown to play critical roles in phospholipid metabolism (Miyata et al. 2018) . The genetic interaction data ( Figure 1a ) is generated from databases that have defined whether two genes are functionally associated specifically through BioGRID (Biological General Repository for Interaction Datasets), which provides a platform to consolidate genetic datasets from various sources. The physical interaction data ( Figure 1b ) identifies if gene products are linked through protein-protein interaction studies and is generated through BioGRID and Pathway Commons, which integrates data from multiple pathway databases (Reactome, KEGG, and BioCarta) to provide a comprehensive view of biological pathways. Through additional analysis using STRING (Search Tool for the Retrieval of Interacting Genes/Proteins; https://string-db.org/) predicted protein-protein interactions and functional associations were explored. Confidence scores are assigned by STRING based on current evidence. The higher the score (closest to 1) the greater the confidence of the interaction/functional association. While the Genemania bioinformatics analysis revealed no current literature reporting a physical interaction between She9 and Mdm31 or Mdm32 ( Figure 1b ), the analysis using STRING indicated possible functional partners meaning the likelihood the proteins are either in direct protein-protein contact, indirectly through sharing a substrate, functioning in the same signaling pathway, or participating in larger multi-protein complexes. The STRING analysis revealed the functional partners for She9 were Mdm31 with a confidence score of 0.838 and with Mdm32 with a confidence score of 0.775. The proteins Mdm31 and Mdm32 were the top-two hits for She9, which is plausible since all three of these proteins localize at the MIM, but further investigation would be required to delineate the exact nature of the relationship.

Additionally, studies have shown that growth in fermentable versus non-fermentable carbon sources can alter phospholipid transport and synthesis in the mitochondria (Miyata et al. 2016) . The nature of this diauxic shift indicates that transition from glycolytic fermentation to respiration can be significant in the understanding of mitochondrial homeostasis. Since phospholipid transport pathways can change dependent on the metabolic state (glycolysis versus oxidative phosphorylation) and since overexpression of *SHE9* has been shown to alter the state of phospholipids CL and PE, the extent to which *she9Δ* would alter mitochondrial phospholipid composition under a non-fermentable carbon source, specifically glycerol, was questioned. It was found that *she9Δ* displays decreased growth on glycerol media (YPG) compared to traditional glucose media (YPD) ( Figure 1c ). Additionally, a temperature shift to 37ºC further exacerbated this defect represented by dilutions 10 ^-4^ and 10 ^-5^ , suggesting that processes other than oxidative phosphorylation limit growth ( Figure 1c ).

While mitochondria are highly dynamic organelles, the growth defect of *she9Δ* could be due to the morphological phenotype causing alterations in the respiratory complexes leading to a compromised ability to produce ATP, therefore hindering cell growth. Alternatively, the growth defect could be due to defects in the maintenance of mitochondrial DNA. Some reports have shown that *she9Δ* also reduces nucleic acid uptake by the mitochondria in *S.cerevisiae* (Weber-Lotfi et al., 2015). This restriction in uptake occurs at the outer mitochondrial membrane, which could support a She9 connection to the Mdm31-Mdm32-Por1 complex. Also, *she9Δ* growth defects could be due to changes in phospholipid metabolism. Whether *she9Δ* results in changes in phospholipid content under non-fermentable carbon source such as glycerol has yet to be investigated. Previous reports indicated *she9Δ* grown in glucose had no change in phospholipid content, but overexpression of *SHE9* was reported to show a decrease in phospholipids CL and PE (Klecker et al. 2015) , The later result suggests a negative regulation of She9 on phospholipids CL and PE (see model Figure 1d ). Previous studies have shown changes in phospholipid synthesis and transport in different carbon-source-medias. Specifically, it has been shown that cells depleted of Ups2-Mdm35 showed little to no effect on the transport of PS and subsequent synthesis of PE when grown in glucose. However, the use of a non-fermentable carbon source causes a diauxic shift, where the cells now prefer respiration to generate ATP over glycolysis. Upon a diauxic shift, the proteins Ups2-Mdm35 are now responsible for approximately half of PE synthesis in the mitochondria (Miyata et al. 2018) . This mechanism is described as the Ups1-independent CL accumulation pathway and in this pathway the protein Por1 interacts with Mdm31/Mdm32/Fmp30 to promote the production of CL (Miyata et al. 2018) . Therefore, previous evidence suggests that a diauxic shift can reveal different molecular functions for proteins.

We propose a model by which under non-fermentable carbon sources, She9 can participate as an inhibitor of the Ups-1 independent CL accumulation pathway ( Figure 1d ). Our hypothetical model suggests that upon the loss of *SHE9* under a non-fermentable carbon source, there would be an overall increase in only PE, since PE has been found to inhibit the production of CL (Miyata et al. 2018) . Increases in PE have been shown to influence membrane fluidity, curvature, and protein-lipid interactions potentially affecting the structure of the mitochondria (Calzada et al. 2016; Klecker and Westermann 2021) . Currently, only our predication analysis through STRING (https://string-db.org/) suggests a relationship between She9 and Mdm31/Mdm32, but the possibility that She9 functions with this complex has yet to be investigated. While this model requires further investigation, our genetic analysis network ( Figure 1a ) reveals reported genes that *SHE9* has been shown to have a functional association, providing a number of potential pathways She9 could be participating in. Regardless, the devastating defects caused by the loss of *SHE9* supports its essential role in proper mitochondrial functioning and homeostasis.

## Methods

*Media and culturing conditions*

*S.cerevisae* strains were purchased from ThermoFisher. Strains were cultured in sterile standard YPD (1% yeast extract, 2% peptone, 2% dextrose (D-glucose) with/without 200mg/L Geneticin (Corning G418; cat#61-234-RG), or YPG (1% yeast extract, 2% peptone, 2% glycerol). For solid agar plates 2% agar was added.

*Spot Assay*

Strains were taken from glycerol stocks and plated on YPD plates (BY4741) and YPD + G418 plates ( *she9Δ)* . To analyze growth phenotypes, single colonies were grown in YPD overnight cultures at 30°C. Cells were harvested by centrifugation and washed twice with sterile distilled water and resuspended in sterile distilled water at an optical density (OD _600_ ) of 1. Serial dilutions were performed to values of 10 ^-1^ , 10 ^-2^ , 10 ^-3^ , 10 ^-4^ , 10 ^-5^ , and 10 ^-6^ . Dilutions were then spotted at 2 µL of suspension per spot on subsequent plates. Strains were spotted in technical duplicates and experiments were repeated as biological triplicates. Plates were grown at 30°C and 37°C respectively for 2-3 days and then imaged. Images were evaluated together and representative images for each plate was selected from all replicates.

*Image Processing*

Fiji ( F iji I s J ust I mageJ) was used to process images of yeast plates. Images were first converted to 8-bit, then the straight-line tool was used to measure the diameter of the largest spot. The largest diameter measurement was then used in the subtract background function through the following commands: click on process, then click on subtract background, then click to set the rolling ball diameter to the recently calculated diameter, then click sliding paraboloid, and lastly click OK. From here brightness and contrast were corrected per image.

*Network Maps*

To investigate genetic and physical interactors of *SHE9,* GeneMANIA (http://genemania.org/) was used. The search bar in the upper left corner allows one to toggle between species, additional functions provided by GeneMANIA that were not used in this analysis are: co-expression, shared protein domains, co-localization, pathway, predicted functional relationships, and other networks that do not fit in the specified categories. The STRING database (www.string-db.org) was used to produce a list of predicted interactors with She9 (Snel et al. 2000; von Mering et al. 2003; von Mering et al. 2005; von Mering et al. 2007; Jensen et al. 2009; Szklarczyk et al. 2011; Franceschini et al. 2013; Szklarczyk et al. 2015; Franceschini et al. 2016; Szklarczyk et al. 2017; Szklarczyk et al. 2019; Szklarczyk et al. 2021; Szklarczyk et al. 2023) .

## Reagents

**Yeast Strains Used in this Study**

**Table:** 

**Name**	**Genotype**	**Reference**
BY4741	*MATa his3-1 leu2-0 met15-0 ura3-0*	(Brachmann et al. 1998)
*she9Δ*	*MATa his3-1 leu2-0 met15-0 ura3-0 SHE9Δ::kanMX4*	(Wach et al. 1994; Winzeler et al. 1999; Giaever et al. 2002)
